# p53 activation: a checkpoint for precision genome editing?

**DOI:** 10.1186/s13073-018-0578-6

**Published:** 2018-08-17

**Authors:** Anastasia Conti, Raffaella Di Micco

**Affiliations:** San Raffaele Telethon Institute for Gene Therapy, Via Olgettina 60, 20132 Milan, Italy

## Abstract

Recent work has reported that the activation of the p53 pathway in primary cells hampers the efficiency of genome editing by CRISPR/Cas9 via induction of apoptosis or cell cycle arrest. These findings highlight the importance of wholly comprehending cellular responses to programmable nucleases in therapeutically relevant cell types to fulfill the promise of genome editing for therapeutic applications for human diseases.

## Genome editing and DNA double-strand breaks

Precision genome editing has the potential to provide novel therapeutic options for many diseases by allowing in-situ correction of inherited mutations or targeted integration of transgene cassettes into safe genomic harbors. The genome-editing arsenal exploits programmable endonucleases, including zinc finger nucleases (ZFNs), transcription activator-like effector nucleases (TALENs), and RNA-based CRISPR/Cas9 nucleases, to induce a DNA double-strand break (DSB) at a pre-defined genomic locus [1]. DSBs are sealed by the error-prone non-homologous end joining (NHEJ) pathway or by the high-fidelity homology-directed repair (HDR) pathway, when a corrected sequence is delivered to the break as a DNA donor template [2]. Therefore, the efficacy of the editing process strictly depends on the DNA repair capacity of the targeted cells and on their susceptibility to the editing procedure. Human pluripotent stem cells (hPSCs), for example, are one of the cell types most resistant to genetic engineering [3]. Recently, two studies [4, 5] reported that even a putative single DSB induced by CRISPR/Cas9 leads to p53-dependent cellular toxicity, ranging from overt apoptosis in hPSCs to cell cycle arrest in an immortalized human retinal pigment epithelial cell line (RPE1). These findings have important implications for the efficacy and safety of gene correction approaches combining PSC technology with genome-editing tools and may affect PSC- and/or RPE-based therapies for monogenic or acquired retinal degenerative diseases.

Ihry et al. [4] used hPSC lines with either stable integration of a doxycycline-inducible Cas9 or transient delivery of pre-assembled ribonucleoprotein complexes (RNPs) for targeted disruption of a panel of genes. Although high efficiency of “indels” was achieved, edited cells showed a significant decrease in their viability. Importantly, this pervasive toxicity was observed not only when editing genes essential for hPSC survival, but also upon editing of transcriptionally inactive genes, dispensable for hPSC growth. Toxicity also did not depend on cellular sensing of the editing machinery as no apoptosis was observed upon administration of an RNP that had been pre-assembled with a non-targeting guide RNA (gRNA). Seeking the mechanisms by which CRISPR/Cas9-induced DSBs trigger apoptosis, the authors found activation of the p53 transcriptional program, a concomitant increase in the levels of the p53 target gene CDKN1A/p21, and induction of physical DNA damage (measured as γH2AX nuclear signal, which accumulates at sites of DSBs). Genetic inactivation of p53 improved the efficiency of hPSC engineering and rescued CRISPR/Cas9-induced toxicity.

**Fig. 1:**
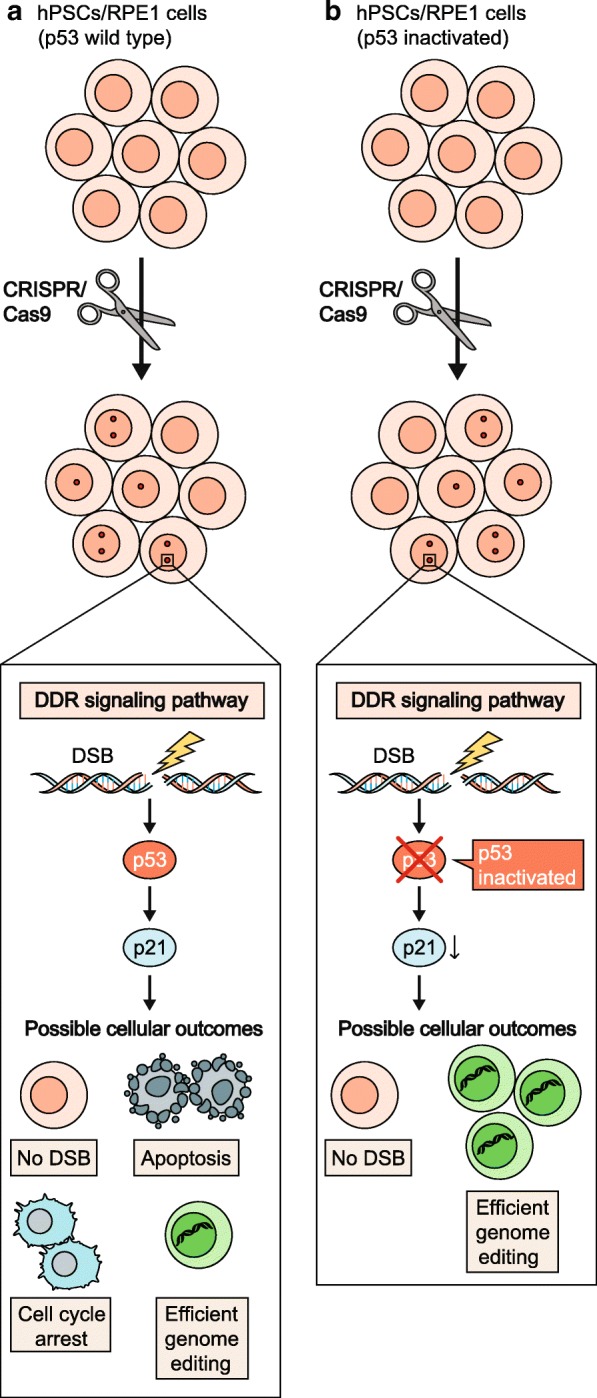
CRISPR/Cas9-induced DSBs cause apoptosis or cell cycle arrest in a p53-dependent manner. Schematic representation of the possible outcomes of CRISPR/Cas9-induced genome editing in p53 wild-type (upper panel) or p53-inactivated (bottom panel) edited cells (human pluripotent stem cells (hPSCs)/human retinal pigment epithelial cells (RPE1)). In p53 wild-type cells, DNA double-strand breaks (DSBs) trigger the activation of the DNA damage response (DDR) pathway with consequent accumulation of p53 and the induction of its target gene p21. Most cells with DSBs undergo apoptosis or cell cycle arrest and only a small number of gene-corrected cells are obtained. p53 genetic inactivation reduces DSB-mediated apoptosis and cell cycle arrest and increases the number of gene-edited cells. No DSBs indicate unedited cells

Similar conclusions were reached by Haapaniemi et al. [5] who conducted a CRISPR/Cas9 screen to identify essential genes in RPE1 cells. In wild-type cells, gRNAs targeting essential genes were not efficiently depleted, while a consistent enrichment for gRNAs targeting cell cycle inhibitors such as p53, p21 and RB1 was observed, indicating that the induction of these genes may limit proliferation of edited cells. Consistent with these observations, the ability of nuclease-treated cells to sustain precision genome editing by homology-driven repair was reduced. Supporting the involvement of p53 in CRISPR/Cas9-induced DNA damage response (DDR) activation and cell cycle arrest, the same screen performed in p53^−/−^ cells led to no enrichment of gRNAs against p21 and to efficient depletion of gRNAs targeting essential genes. The authors also described a modest but significant increase in the editing efficiency in p53-inactivated cells (Fig. 1).

## p53 activation in genome-editing strategies

The tumor suppressor p53 is the most potent cell cycle checkpoint that preserves genome stability by triggering cell cycle arrest, cellular senescence, and/or apoptosis in response to DNA-damaging insults [6]. Therefore, it may not be surprising that cellular response to CRISPR/Cas9 editing, which is reliant on DSBs, involves p53 pathway activation. Others have already reported that multiple DSBs induced by Cas9 can cause cell death in transformed cells [7], but what is striking about these recent studies is the claim that even a single DSB can induce massive cell death in hPSCs and can cause proliferative disadvantage in RPE1 cells to an extent that precludes HDR efficiency. Given the numerous examples of successful genome editing in p53 wild-type cells, one would have indeed anticipated that transient delivery of highly optimized reagents with no reported off-target cleavage would induce only a modest and transient p53 pathway activation, without any detectable impact on cell function. Although p53-mediated cell cycle arrest was observed in RPE1 cells, the exacerbated p53-mediated toxicity to even low levels of Cas9-induced DSBs reported by Ihry et al. [4] may be a unique feature of hPSCs, and more mechanistic studies are urgently needed to assess whether the reported findings represent a general phenomenon of stem cell response to nuclease-induced DSBs compared with the responses of more differentiated cell types. Because edited cells rely on endogenous pathways to repair DSBs, the observed toxicity in hPSCs may reflect reduced expression levels or delayed kinetics of DSB repair pathway activation that could in turn lead to prolonged engagement of the p53 pathway. Consistent with possible faulty repair in this cell type, recent work in murine embryonic stem cells (ESCs) revealed a high frequency of large deletions and complex chromosomal rearrangements due to the repair of DSBs induced by CRISPR/Cas9 [8].

In both studies, stable inactivation of p53 rescued cellular viability and efficiency of precision genome editing in the presence of a DNA donor template. While suppression of p53 may be a valuable approach to increase editing efficiency for basic research purposes, its constitutive inactivation may unleash the proliferation of edited cells, increase their mutational burden and chromosomal rearrangements, and pose a risk of potentially oncogenic events. Consequently, temporary p53 inhibition may represent a safer and more sensible strategy for efficient genome editing, while limiting any potential detrimental effect due to its permanent loss.

If PSCs are generally more sensitive to DSBs, one would predict that other genome-editing platforms acting through induction of DSBs, including ZFNs or TALENs, may elicit a similar biological response. To determine this, studies comparing and contrasting cellular responses to DSBs at the same genomic locus using different genome-editing platforms should be performed. The previously reported increased retention of Cas9 on DNA ends and the slower repair rates highlighted by mathematical modeling of Cas9-induced DSBs [9] could also contribute to amplifying the cellular response to even the few DSBs observed in PSCs.

## Implications for the future of therapeutic gene editing

No clinical trials have yet been conducted with genome-edited hPSCs or their differentiated progeny; moreover, concern that edited hPSCs may be selected for inactivating mutations of p53 or members of its pathway poses new challenges for the prospect of gene-corrected PSC-based cell replacement therapies. Evidence for the safety and long-term stability of edited cells via therapeutic use of T cells edited by ZFNs has been provided by a clinical trial with a follow-up period of almost a decade (ClinicalTrials.gov: NCT01044654). Similarly, autologous hematopoietic stem cells (HSCs) edited ex vivo by ZFN technology have entered phase I clinical trials for patients with transfusion-dependent beta-thalassemia and patients infected with HIV (ClinicalTrials.gov: NCT03432364 and NCT02500849, respectively); other HSC-based CRISPR/Cas9 clinical trials for immune-hematological deficiencies are soon to be launched.

Although stringent evaluation of the p53-dependent DDR to nuclease-induced DSBs in these clinically relevant stem cell sources has yet to be performed, the positive results of the clinical trials to date imply that the response in HSCs may be more contained than that observed in PSCs. The likely different sensitivity to nuclease-induced DSBs in these two stem cell types may be explained by increased DSB repair proficiency in HSCs versus PSCs, as well as by different cell cycle kinetics, given that long-term repopulating HSCs are mainly dormant while PSCs are actively cycling and may face a higher DSB burden due to increased DNA replication stress. Strategies aimed at selectively increasing the activity of HDR-mediated repair factors over NHEJ are emerging as powerful tools to improve genome-editing efficiency in difficult-to-edit cell types. These approaches may particularly benefit PSCs by reducing exposure time of edited cells to unrepaired DNA lesions, preventing induction of the p53 pathway and thus preserving PSC viability. However, careful evaluation of the risks associated with modulation of DNA repair pathways should be performed, given that even a transitory window of defective DSB repair concomitant with suboptimal culture conditions may contribute to increased PSC genomic instability.

These new findings have had a far-reaching impact not only within the scientific community, but also raising public awareness of the potential adverse effects of genome editing, notably depreciating the market value of several biotechnology companies developing genome editing for clinical applications. Media coverage of this work has incidentally emphasized the possible tumorigenic risk associated with genome-editing procedures in a way that could jeopardize its therapeutic potential. However, these conclusions were likely misinterpreted extrapolations from the two studies, as the authors describe induction rather than loss of p53 upon nuclease-induced DSBs and neither study showed evidence supporting causality between CRISPR/Cas9 editing and selection of p53-inactivating mutations.

It remains to be investigated whether emerging non-DSB-inducing genome-editing technologies, such as Cas9-derived base-editing (BE) platforms that provide precise editing at a single base-pair resolution without DNA cleavage [10], similarly trigger a p53-mediated cellular response. BE platforms have not yet passed preclinical safety for therapeutic application tests. However, if proven less harmful, BE platforms may represent a viable alternative to DSB-inducing nucleases for basic research and screening approaches. More generally, these studies encourage investigation of unintended consequences of genome-editing procedures and risk/benefit assessments for each type of target cell and given disease. These findings also further emphasize that a thorough mechanistic understanding of cellular functions is needed to ensure the progress and success of genome-editing-based therapies.

## Abbreviations

BE: Base editing
Cas9: CRISPR-associated protein 9
CRISPR: Clustered regularly interspaced short palindromic repeats
DDR: DNA damage response
DSB: double-strand break
ESC: Embryonic stem cells
gRNA: Guide RNA
HDR: Homology-directed repair
hPSC: Human pluripotent stem cell
HSC: Hematopoietic stem cell
NHEJ: Non-homologous end joining
RNP: Ribonucleoprotein complex
RPE1: human retinal pigment epithelial cells
TALEN: transcription activator-like effector nucleases
ZFN: Zinc finger nuclease
